# Ameliorative Effect of Zinc Oxide Nanoparticles on Antioxidants and Sperm Characteristics in Streptozotocin-Induced Diabetic Rat Testes

**DOI:** 10.1155/2015/153573

**Published:** 2015-10-25

**Authors:** Mohamed Afifi, Omar A. Almaghrabi, Naif Mohammed Kadasa

**Affiliations:** ^1^Department of Biological Sciences, Faculty of Science, University of Jeddah, Jeddah, Saudi Arabia; ^2^Department of Biochemistry, Faculty of Veterinary Medicine, Zagazig University, Zagazig, Egypt

## Abstract

The present study investigated the impact of zinc oxide nanoparticles (ZnONPs) on the oxidative status and sperm characteristics in diabetic rat testicular tissue. Forty male albino rats were used in this study; 10 of them served as a control and 30 rats were injected with a single dose (100 mg/kg) of streptozotocin intraperitoneally. They were subdivided into diabetic, diabetic + ZnONPs (10 mg/kg B.W.), and diabetic and cotreated with ZnONPs + insulin groups. The sperm count and motility were assessed. The activity and mRNA expression of superoxide dismutase (SOD), catalase (CAT), glutathione peroxidase (GPx), glutathione reductase (GRD), and Glutathion-S-Transferase (GST) were determined in the testicular tissue. Malondialdehyde (MDA) and reduced glutathione (GSH) levels were estimated in the testicular tissue. Sperm count and motility increased in ZnONPs treated diabetic rats. A significant increase in the activity and mRNA expression of SOD, CAT, GPx, GRD, and GST was shown in ZnONPs treated diabetic rats. MDA significantly decreased, while GSH increased in testicular tissue of ZnONPs treated diabetic rats. It was concluded that ZnONPs either alone or in combination with insulin have the ability to increase the sperm count and motility and protect the testicular tissue against the oxidative stress induced by diabetes in rats.

## 1. Introduction

Diabetes mellitus is a group of metabolic diseases characterized by increase of blood glucose level [1]. The large patient numbers and the consequences of the disease push many scientists to develop several therapies which are costly to a large limit. The most common consequence of diabetes mellitus is the generation of reactive oxygen species (ROS) [2]. ROS can induce *β*-cell failure and develop insulin resistance [3]. Diabetes mellitus can directly affect male fertility through the generation of an oxidative stress. The subfertility prevalence rate was high (51%) among diabetic patients [4]. It is well known that DM induces an adverse change in sperm number, quality, and function [5]. Additionally, DM adversely affects male fertility at many levels, including ejaculation, endocrine control of spermatogenesis, erection, semen volume, and spermatozoa vitality and motility [6]. Type 1 diabetic adolescents had semen of low volume, motility, altered morphology, and high fructose and glucose levels, indicating that ineffective metabolic control was the cause of these alterations [7]. The key mechanism is illustrated as an increase in mitochondrial glucose oxidation which was induced by hyperglycemia, releasing a huge amount of superoxide and other free radicals into the cytoplasm [8]. The recent reports mentioned that the prolonged hyperglycemia produces advanced glycation end-products (AGE) which induce superoxide generation [9]. Zn has been reported to play a direct role in glucose homeostasis through enhancing hepatic glycogenesis through its actions on the insulin signaling pathway and thus it improves glucose utilization [10]; it inhibits intestinal glucose absorption [11] and increases glucose uptake in skeletal muscle and adipose tissue [10]. Moreover, Zn is reported to inhibit glucagon secretion [12], thus reducing gluconeogenesis and glycogenolysis; it also enhances the structural integrity of insulin [13]. Decreased Zn in the pancreas may reduce the ability of the islet *β*-cells to synthesize and lead insulin in the blood [14]. Furthermore, knowing zinc's antioxidant role [15], reduced Zn may exacerbate the oxidative stress-mediated complications of diabetes. The pivotal role of Zn in diabetes mellitus was discovered by supplementation studies in diabetic rats [16]. Several Zn complexes have been synthesized and proven to be effective in rodent models of diabetes [17]. Recently there has been a huge development of nanotechnology in the science and technology field; metallic nanoparticles, like gold, silver, iron, Zn, and metal oxide nanoparticles, have shown great challenges in the field of medicine and its applications [18]. In a previous study, we proved the antidiabetic effect of ZnONPs and SNPs as a novel agent to control diabetes mellitus in rats [19]. The aim of the study is to test the ability of ZnONPs to reduce the oxidative stress induced by diabetes mellitus in testicular tissues of diabetic rats and study the possible ameliorative effects of ZnONPs against diabetes-related sperm quality.

## 2. Materials and Methods

### 2.1. Animals

Forty male albino (Sprague-Dawley) rats with average age and weight at the beginning of the experiment (20 ± 2 weeks and 120 ± 20 g) were used in the study. Rats were housed with each other in the animal house at the Faculty of Science, King Abdulaziz University, for 7 days before the start of the experimental procedures. All rats were grouped into four groups; the first one is control (*N* = 10); it was not subjected to any treatment. The remaining thirty animals were induced to be diabetic through intraperitoneal injection of a single dose (100 mg/kg) of streptozotocin (STZ) supplied by Sigma-Aldrich (Catalog no. S0130 SIGMA, Germany). The diabetic rats were further divided into four groups; rats of diabetic group (*N* = 10) served as a positive control with no treatment; rats of diabetic + ZnONPs group were administered 10 mg/kg/day of ZnONPs per OS; and rats of diabetic + ZnONPs + insulin group were administered a daily dose of ZnONPs (10 mg/kg) and were injected subcutaneously with insulin in a dose of 1.2 units/100 g/day for 30 constitutive days [19].

### 2.2. Ethical Statement

All experimental procedures were performed in agreement with King Abdulaziz University's experimental animal ethics.

### 2.3. Diabetes Mellitus Induction

DM was induced by a single intraperitoneal injection of STZ (100 mg/kg) [19]. Rats were fed on a rat formulated diet {basal rat diet (65.5%), sucrose (20%), and margarine (10%)}. Fasting blood glucose was monitored in the blood of the injected rats after 48 h from the injection. Rats of fasting blood glucose level more than 300 mg/dL were used as diabetic rats.

### 2.4. Sampling Protocol

Rats were anesthetized with ketamine and xylazine (150 mg/kg and 10 mg/kg, resp.); testes were collected from animals of all experimental groups. The testicular tissue was divided into different aliquots which were preserved at −80°C until their use in biochemical and molecular biological investigations.

### 2.5. Epididymal Sperm Preparation and Analysis

Rats were anesthetized with ketamine and xylazine (150 mg/kg and 10 mg/kg, resp.). The code epididymis was dissected and placed in 1 mL of prewarmed Ham's F10 medium for 30 min. Gentle tearing was done to swim out spermatozoa into the culture media. Sperm count (10^6^/mL) and motility were assessed using Makler chamber [20].

### 2.6. Biochemical Assay

Testicular tissue GSH, CAT, SOD, GPx, GR, and GST activities were determined using the kits (Catalog nos. NWK-GSH01, NWK-CAT01, NWK-SOD01, NWK-GPX01, NWK-GR01, and NWK-GST01) purchased from Northwest Life Science Specialties (NWLSS), Vancouver, Canada. MDA was analyzed by measuring the production of thiobarbituric acid reactive substances (TBARS) using TBARS assay kit (Catalog no. 10009055, Cayman, USA).

### 2.7. Molecular Analysis

Testicular tissue SOD, CAT, GPx, GRD, and GST gene expressions were quantified using real time PCR. Total RNA was isolated from tissue samples using the RNeasy Mini Kit Qiagen (Catalog no. 74104). 0.5 *μ*g of total RNA was used for production of cDNA using Qiagen Long Range 2 Step RT-PCR Kit (Catalog no. 205920). Five *μ*L of total cDNA was mixed with 12.5 *μ*L of 2x SYBR Green PCR mix with ROX from BioRad and 10 pmol/*μ*L of each forward and reverse primer for the measured genes. The house keeping gene *β*-actin was used as a constitutive control for normalization. Primers were designed using Primer3 software (http://bioinfo.ut.ee/primer3/) as per the published rat SOD, CAT, GPx, GRD, and *β*-actin gene sequences (Table 1) of NCBI database; all primaries were provided by Sigma-Aldrich (Sigma-Aldrich Chemie GmbH, Steinheim, Germany). PCR reactions were carried out in ABI PRISM 7300 (Applied Biosystems, USA). The RNA concentration in each sample was determined from the threshold cycle (Ct) values. The mRNA expression levels were calculated relative to *β*-actin gene's mRNA levels using 2^−DD CT^ method.

### 2.8. Statistical Analysis

The obtained data were analyzed using SPSS version 20 (IBM, 1 New Orchard Road, Armonk, New York 10504-1722, United States). Data were presented as mean ± SD (*N* = 10). Comparison among groups was made by one-way analysis of variance (ANOVA). Duncan's test was used for testing the intergrouping homogeneity. Statistical significance was set at *p* < 0.05.

## 3. Results

### 3.1. Effect of ZnONPs on Sperm Count and Motility

Effect of ZnONPs on sperm count and motility was shown in Table 2. Our result reported that the sperm count was 46.9% lower in the diabetic group than in control rats. The total sperm count in the diabetic group was 57.5 ± 4.5 million/mL, which was significantly lower than the control count (108.3 ± 5.7 million/mL). Thirty-day treatment with ZnONPs at 10 mg/kg/day produced a significantly increased count (38.5%) as compared to diabetic rats. Administration of ZnONPs + insulin produced 41.8% higher count when compared to diabetic rats. No significant changes in the sperm count were investigated between rats administrated ZnONPs alone or in combination with insulin. The grade a and total sperm motility in diabetic rats were 86.5 and 40.3% lower than those in normal control rats. Meanwhile the immotile sperm (grade d) in the diabetic group was 62.4% higher than that in the control group. Thirty-day treatment with ZnONPs produced a significantly higher forward and total sperm motility (83.8% and 36.4%, resp.) when compared to the diabetic group. Meanwhile ZnONPs + insulin produced 85.7 and 39.6% higher progressive and total sperm motility when compared to the diabetic group. ZnONPs + insulin were more effective than ZnONPs alone in increasing the progressive and total sperm motility in diabetic rats.

### 3.2. Effect of ZnONPs on Testosterone Levels in Diabetic Rats

Our result reported that the serum testosterone level decreased in diabetic rats as compared with control rats. Administration of ZnONPs to the diabetic rats for 30 days resulted in a significant increase in the serum testosterone level when compared to the diabetic nontreated group. Serum testosterone levels in diabetic treated groups returned significantly to approximate levels of the control group. Treatment with ZnONPs alone or in combination with insulin approximated serum testosterone levels as the control.

### 3.3. Effect of ZnONPs on Testicular Tissue MDA Levels

Our result reported that (Table 3) the MDA concentration in testicular tissue of the diabetic rats was 4.7 times higher than that in control nondiabetic rats. Thirty-day treatment with ZnONPs at 10 mg/kg/day caused a significant decreased MDA concentration (69.6%) when compared to diabetic rats. Administration of ZnONPs + insulin to the diabetic rats produced 76.8% decreased MDA level when compared to diabetic rats.

### 3.4. Effect of ZnONPs on Testicular Tissue Antioxidant Gene Expression

Our result showed that the mRNA levels of SOD, CAT, GPx, GR, and GST in testicular tissue of the diabetic rats were significantly lower than those in control nondiabetic rats. Thirty-day treatment with ZnONPs at 10 mg/kg/day caused a significant increase of SOD, CAT, GPx, GR, and GST mRNA expression levels when compared to the diabetic group. ZnONPs + insulin were more effective than ZnONPs alone in increasing the SOD, CAT, GPx, GR, and GST mRNA expression levels in diabetic rats (Table 4).

### 3.5. Effect of ZnONPs on Testicular Tissue Antioxidant Enzyme Activities

Our result showed that (Table 3) the GSH level and SOD, CAT, GPx, GR, and GST activities in testicular tissue of the diabetic rats were significantly lower than those in control nondiabetic rats. Thirty-day treatment with ZnONPs at 10 mg/kg/day caused a significant increase of GSH level and SOD, CAT, GPx, GR, and GST activities when compared to the diabetic group. ZnONPs + insulin were more effective than ZnONPs alone in increasing the GSH level and SOD, CAT, GPx, GR, and GST activities in diabetic rats.

## 4. Discussion

This study investigated the effect of ZnONPs on sperm count, motility, and oxidative stress in testicular tissue of the diabetic rats. Sperm analysis is useful for determination of the male infertility cause [21]. Our results reported that sperm count and motility lowered in diabetic rats, while the dead sperm increased. Many mechanisms explained this finding including the hormonal impairment of follicle-stimulating hormone (FSH), luteinizing hormone (LH), and testosterone. The oxidative stress induced by hyperglycemia and formation of advanced glycation end-products (AGE) was incriminated in the bad effect of diabetes on sperm [22]. The effect of diabetes on sperm count and motility was consistent with other investigations in both humans and rats [23]. Short-term hyperglycemia was adversely affecting the sperm count in diabetic rats [24]. The oxidative stress was incriminated as the cause of the bad effect of prolonged hyperglycemia on sperm count and motility in diabetic rats [25]. The present study proved that treatment of the diabetic rats with ZnONPs alone or in combination with insulin prevented damage in sperm count and motility; this may be due to the antioxidant characters of ZnONPs [26]. Treatment with ZnONPs produced an increased sperm count, proving that ZnONPs have the ability to protect the sperm from the deleterious effect induced by diabetes. ZnONPs + insulin were more effective than ZnONPs alone in increasing the progressive and total sperm motility in diabetic rats. The effect of insulin on sperm characteristics was previously documented; insulin and/or glucose effect on the pituitary biosynthesis and/or secretion of FSH was necessary for spermatogenesis [22].

Our result reported that the serum testosterone level was lower in diabetic rats than in control nondiabetic rats. In the same line, Ding et al. [27] showed that men with diabetes had significantly lower levels of serum testosterone when compared with men without diabetes. Brand et al. [28] have shown that diabetic men had not only lower testosterone but also lower levels of sex hormone binding globulin (SHBG) when compared with nondiabetic men. Potential mechanisms for low testosterone levels in type 2 diabetes mellitus include reduced or absent stimulatory effect of insulin on Leydig cells [29], increased leptin levels in diabetes causing Leydig cell dysfunction [30], and increased TNF levels in diabetes inhibiting steroid biosynthesis in Leydig cells [31]. Our finding proved the ability of ZnONPs alone or in combination with insulin to approximately alleviate the bad effect of diabetes on serum testosterone levels. Potential mechanisms for induction of testosterone production in rats administrated ZnONPs include increased insulin production and sensitivity as proved by our previous work [19]. Zn increased the release of luteinizing and follicle stimulating hormones from the pituitary gland, which stimulate testosterone production; Zn also inhibits the aromatase enzyme that converts testosterone into excess estrogen [32].

The present investigation pointed to a reduction in GSH levels and SOD, CAT, GPx, GR, and GST activities and gene expressions; meanwhile, the MDA level was increased in diabetic nontreated rats. It is well known that diabetes mellitus has the ability to induce an oxidative stress in many tissues [33]. The increase in mitochondrial glucose oxidation induced by hyperglycemia releases a huge amount of superoxide and other free radicals into the cytoplasm [8]. The recent reports mentioned that the prolonged hyperglycemia produces AGEs, which is the cause of superoxide generation [9]. This activates NADPH oxidase [34], which increases superoxide generation [35]. Diabetes induced a state of oxidative stress, which was evidenced by an increase in levels of MDA and a decrease in SOD and GST activities in testicular tissues [22].

Our findings have further shown that ZnONPs were able to decrease the oxidative stress in testicular tissue as manifested from decreased MDA levels, higher GSH amount, and induced antioxidant enzymes (SOD, CAT, GPx, GR, and GST) activities and gene expressions in the testicular tissue of diabetic rats. We proved the antioxidant ability of ZnONPs in diabetic rat brain tissue. It increased the activity and mRNA expression levels of SOD, CAT, GRD, and GPx and GSH levels and decreased MDA levels in brain tissue of the treated diabetic rats [26]. Potential mechanisms for antioxidant ability of ZnONPs may include the potent antidiabetic effect of ZnONPs; it induced significantly reduced blood glucose, increased serum insulin, and activated glucose oxidation preventing hyperglycemia in diabetic rats [19], which decreases superoxide generated by advanced glycation end-products [9]. The other mechanism may be the increased Zn concentration in testicular tissue produced from the dissociation of ZnONPs. It is well known that Zn is a powerful antioxidant metal; it is the core constituent of antioxidant enzymes such as SOD and a recognized protector of sulfhydryl groups; it is also thought to impair lipid peroxidation by displacing transition metals such as iron and copper from catalytic sites [36]. ZnONPs is able to protect cell membrane integrity against oxidative stress damage, increase antioxidant enzyme levels, and decrease MDA level. It can improve antioxidant activity, enhance the activities of antioxidases, and decrease the levels of free radicals [37].

In conclusion, DM has an adverse effect on the antioxidant status of the testicular tissue through induction of lipid peroxidation as represented by increased MDA level; it decreased the gene expression and activity of the antioxidant enzymes such as SOD, CAT, GDR, and GPx. ZnONPs alone or in combination with insulin improved the antioxidant status of the testicular tissue and improved some sperm parameters in diabetic rats.

## Figures and Tables

**Table 1:** Primers oligonucleotide sequences of SOD, CAT, GPx, GR, and *β*-actin genes.

Gene	Oligonucleotides sequences		Size (bp)	Gene ID
SOD	F	5′-TCACTTCGAGCAGAAGGCAA-3′	221	NM_017050.1
	R	5′-CCTTTCCAGCAGCCACATTG-3′		

CAT	F	5′-ATGGCTATGGCTCACACACC-3′	507	NM_012520.2
	R	5′-GAGCACGGTAGGGACAGTTC-3′		

GPx	F	5′-GCTCACCCGCTCTTTACCTT-3′	387	M21210.1
	R	5′-AATGGACGAGAACATGCCCA-3′		

GRD	F	5′-CCATGTGGTTACTGCACTTCC-3′	171	NM_053906
	R	5′-GTTCCTTTCCTTCTCCTGAGC-3′		

GST	F	5′-AGCCCTGATTGACATGTACACCG-3′	284	NM_017013.4
	R	5′-AGGCCTTCAGCAGAGGGAAA-3′		

*β*-actin	F	5′-TCACTATCGGCAATGTGCGG-3′	260	NM_007393
	R	5′-GCTCAGGAGGAGCAATGATG-3′		

**Table 2:** Effect of zinc oxide nanoparticles and insulin on sperm analysis and serum testosterone level in streptozotocin-induced diabetic rat.

Parameters	Control	Diabetic	Diabetic + ZnONPs	Diabetic + ZnONPs + insulin
Count (million/mL)	108.3 ± 5.7^a^	57.5 ± 4.5^c^	93.5 ± 9.3^b^	98.8 ± 3.8^ab^
Rapid motility (%) (grade a)	44.45 ± 3.6^a^	6 ± 0.05^c^	37.1 ± 2.8^b^	42 ± 4.4^a^
Slow motility (%) (grade b)	13.55 ± 0.5^b^	18.7 ± 2.6^a^	15 ± 1.3^b^	15 ± 0.8^b^
Nonprogressive motility (%) (grade c)	22.4 ± 5.7	23 ± 2.2	23 ± 2.8	22 ± 1.6
Immotile sperm (%) (grade d)	20 ± 1.6^b^	53.3 ± 7^a^	24.9 ± 2^b^	21 ± 2^b^
Motility (%)	80 ± 9.8^a^	47.7 ± 4.9^c^	75.1 ± 6.9^b^	79 ± 6.8^a^
Serum testosterone (ng/dL)	5.7 ± 1.6^a^	2.6 ± 0.8^b^	5 ± 1.6^a^	6.2 ± 1.4^a^

Each point represents the mean ± *D* from triplicate determinations (*n* = 10 rats per group). Means within the same row carrying different superscripts (a, b, and c) are significant at *p* < 0.05.

**Table 3:** Biochemical investigations in experimental rats administered zinc oxide nanoparticles.

Parameters	Control	Diabetic	Diabetic + ZnONPs	Diabetic + ZnONPs + insulin
MDA (nmol/g tissue)	1.2 ± 0.06^b^	5.6 ± 0.5^a^	1.7 ± 0.3^b^	1.3 ± 0.05^b^
GSH (*μ*mol/g tissue)	123 ± 4^ab^	40 ± 2^c^	121 ± 8^b^	131 ± 4^a^
SOD (*μ*g/g tissue)	55.7 ± 3.2^a^	24 ± 2.6^c^	47.3 ± 2.5^b^	56.7 ± 3.5^a^
CAT (*μ*MH_2_O_2_ decomposed/g tissue)	1043 ± 51^a^	223 ± 25^c^	943 ± 40^b^	1110 ± 36^a^
GPx (*μ*M/min/g tissue)	166.7 ± 15^a^	35.7 ± 5^b^	161 ± 7^a^	173 ± 12^a^
GRD (unit/g tissue)	15.7 ± 2^ab^	2.9 ± 0.9^c^	13 ± 2^b^	16.1 ± 0.8^a^
GST (*μ*mol GSHCDNB conjugate/min/mg protein)	86 ± 3.5^ab^	26 ± 4^c^	81 ± 6^b^	92 ± 2.5^a^

Each point represents the mean ± *D* from triplicate determinations (*n* = 10 rats per group). Means within the same row carrying different superscripts (a, b, and c) are significant at *p* < 0.05.

**Table 4:** Effect of zinc oxide nanoparticles on the mRNA expression profile (fold of change relative to the control group) of antioxidant genes in streptozotocin-induced diabetic rat testes.

Genes	Control	Diabetic	Diabetic + ZnONPs	Diabetic + ZnONPs + insulin
SOD	1 ± 0.01^a^	0.15 ± 0.02^b^	0.99 ± 0.09^a^	1.12 ± 0.15^a^
CAT	1.07 ± 0.02^b^	0.22 ± 0.02^c^	1.04 ± 0.02^b^	1.7 ± 0.25^a^
GPx	1.06 ± 0.02^b^	0.30 ± 0.09^c^	1.06 ± 0.02^b^	3.3 ± 0.87^a^
GR	1.05 ± 0.03^b^	0.29 ± 0.08^c^	1.4 ± 0.26^b^	4 ± 0.6^a^
GST	0.94 ± 0.03^b^	0.13 ± 0.02^c^	1.06 ± 0.03^b^	1.76 ± 0.4^a^

Each point represents the mean ± *D* from triplicate determinations (*n* = 10 rats per group). Means within the same row carrying different superscripts (a, b, and c) are significant at *p* < 0.05.
